# Tolerance Even to Lethal Strain of Potato Spindle Tuber Viroid Found in Wild Tomato Species Can Be Introduced by Crossing

**DOI:** 10.3390/plants10030575

**Published:** 2021-03-18

**Authors:** Takashi Naoi, Tatsuji Hataya

**Affiliations:** 1 Pathogen-Plant Interactions, Graduate School of Agriculture, Hokkaido University, Kita 9, Nishi 9, Kita-ku, Sapporo 060-8589, Japan; naoi@res.agr.hokudai.ac.jp; 2 Pathogen-Plant Interactions, Research Faculty of Agriculture, Hokkaido University, Kita 9, Nishi 9, Kita-ku, Sapporo 060-8589, Japan

**Keywords:** potato spindle tuber viroid, tomato, wild tomato species, tolerance, F1 hybrid, crossing and breeding

## Abstract

To date, natural resistance or tolerance, which can be introduced into crops by crossing, to potato spindle tuber viroid (PSTVd) has not been reported. Additionally, responses to PSTVd infection in many wild tomato species, including some species that can be crossed with PSTVd-susceptible cultivated tomatoes (*Solanum lycopersicum* var. *lycoperaicum*), have not been ascertained. The aim of this study was to evaluate responses to PSTVd infection including resistance and tolerance. Accordingly, we inoculated several cultivated and wild tomato species with intermediate and lethal strains of PSTVd. None of the host plants exhibited sufficient resistance to PSTVd to render systemic infection impossible; however, these plants displayed other responses, including tolerance. Further analysis of PSTVd accumulation revealed low accumulation of PSTVd in two wild species, exhibiting high tolerance, even to the lethal strain. Additionally, F1 hybrids generated by crossing a PSTVd-sensitive wild tomato (*Solanum lycopersicum* var. *cerasiforme*) with these wild relatives also exhibited tolerance to the lethal PSTVd strain, which is accompanied by low PSTVd accumulation during early infection. These results indicate that the tolerance toward PSTVd in wild species is a dominant trait and can be utilized for tomato breeding by crossing.

## 1. Introduction

Viroids are single-stranded (ss) circular RNA molecules ranging from 246 to 401 nucleotides (nt) in length and are known as the smallest plant pathogens [1,2]. They do not encode any proteins in their genome sequences; hence, they autonomously replicate depending on host plants’ transcriptional machinery [3,4]. Their highly base-paired stem-loop structures and partially double-stranded (ds) replicative intermediates that form transiently during replication are potential targets of the host RNA silencing mechanism [5] and are processed by Dicer-like (DCL)—an RNase III-like enzyme—into 21–24 nt viroid-derived small RNAs (vd-sRNAs). Vd-sRNAs are recruited by the RNA-induced silencing complex (RISC) including Argonaut, which is an RNase H-like enzyme. RISC directs the cleavage of complementary target viroid RNA according to the incorporated vd-sRNA sequence. The vd-sRNAs in RISC also suppress host gene expression with complementary sequences to the vd-sRNAs and are involved in disease-related symptom expression of the viroids [6,7,8,9]. These reports suggest a close relationship between RNA silencing and symptom expression in viroid diseases.

Potato spindle tuber viroid (PSTVd), a type species of the genus *Pospiviroid* and one of the most studied viroids, has a wide host range and generally infects Solanaceae and Asteraceae plants. In particular, cultivated tomatoes (*Solanum lycopersicum* var. *lycopersicum*, *S. l. lycopersicum*) are the most prominent host plants for pospiviroids, including PSTVd. PSTVd isolates are classified into four strains (mild, intermediate, severe, and lethal) depending on their pathogenicity as indicated by the degree of disease symptoms in ‘Rutgers’ tomatoes [10]. This difference in pathogenicity with the same host indicates that the PSTVd genome sequence itself is one of the pathogenic determinants. For example, mutations of 4 or 3 nt transformed the intermediate PSTVd isolate Int into lethal isolates AS1 or RG1, in ‘Rutgers’ tomatoes [11,12].

The high sensitivity of the tomato cultivar ‘Rutgers’, which was originally used as an indicator plant for potato witches’ broom disease [13], to PSTVd infection was found before the first report on viroid diseases [14]. Since Diener in 1971 reported the causative pathogen of potato spindle tuber disease as the potato spindle tuber “viroid”, ‘Rutgers’ tomatoes have been used as a sensitive host and an indicator plant in a wide range of studies concerning PSTVd [15]. Recently, a highly tolerant tomato cultivar ‘Moneymaker’, exhibiting only mild symptoms of PSTVd infection, has been used for comparison with the highly sensitive ‘Rutgers’ in studies of viroids regarding the mechanism of disease symptom expression and anti-viroid defense responses [16,17]. The PSTVd symptoms and sensitivity vary by tomato cultivar, suggesting the role of host factors; however, the relationship between the pathogenicity determinants in viroid and host factors involved in symptom expression is largely unknown.

Indigenous South American populations commenced early domestication of edible wild tomato berries in the Andes region, where the majority of the wild tomato *S. lycopersicum* var. *cerasiforme* (*S. l. cerasiforme*, so-called cherry tomatoes) and 12 native wild tomato relatives (*S. pimpinellifolium, S. cheesmaniae, S. galapagense, S. chmielewskii, S. arcanum, S. neorickii, S. huaylasense, S. pervianum, S. corneliomulleri, S. chilense, S. habrochaites*, and *S. pennellii*) have evolved [18,19]. Among these wild tomato species, *S. l. cerasiforme* and *S. pimpinellifolium* are considered the ancestors of cultivated tomatoes [18,20,21,22]. Since these wild tomato species bear small fruits, it is supposed that during domestication and subsequent breeding, tomatoes accumulated mutations in the genome that increased their fruit size until they reached the size of currently cultivated tomatoes [18,22,23,24].

Wild tomatoes can be crossed with cultivated tomatoes to introduce useful traits, such as tolerance to stress and resistance to pathogens. For example, *Tm-1*, a semi-dominant resistance gene of tomatoes to tomato mosaic virus (ToMV), was introgressed from *S. habrochaites* and has been used to protect tomato plants against ToMV infection [25]. In addition, whole-genome sequence analysis of *S. pennellii* revealed that the wild tomato relative has useful genes for tomato breeding, such as those promoting stress tolerance, that may have been lost during the process of tomato cultivation [26].

Some wild relatives of tomatoes were tested for susceptibility to PSTVd in early studies to investigate the experimental host range of PSTVd. It was reported that *S. l. cerasiforme* and *S. pimpinellifolium* are asymptomatic, whereas *S. pervianum*, *S. habrochaites*, and *S. cornellionulerii* are symptomatic during PSTVd infection [27,28]. Thus, similar to tomato cultivars, wild tomato species are presumed to present various responses to PSTVd infection, but this has not been confirmed in most of the species.

To date, *Chrysanthemum morifolium* and chrysanthemum stunt viroid (CSVd) represent the only host–viroid pair for which several surveys on viroid resistance have been carried out [29]; however, the inheritance of CSVd resistance remains unclear. When the resistance cultivar ‘Okayama Heiwa’ was crossed as the female parent with two susceptible cultivars, ‘Sei Elza’ and ‘Anri’, which were the pollen parents, only a portion of the first filial generation (F1) plants demonstrated CSVd resistance [29,30]. For PSTVd, two lines of wild potatoes (*Solanum berthaultii*) were reported to be resistant to viroid sap inoculations [31]. Furthermore, PSTVd resistance or tolerance has been reported in other wild potato relatives [32,33]; however, the resistance traits have not been transferred to cultivated potatoes (*Solanum tuberosum*) [33,34]. Thus, PSTVd resistance that can be introduced into susceptible cultivated crops by crossing has not been reported; however, tolerance is the only available phenotype for general cross-breeding to generate cultivars suppressing PSTVd disease. Meanwhile, in a previous study, the symptomatic trait was dominant during PSTVd-AS1 infection in the F1 hybrid obtained by crosses between PSTVd-tolerant and -sensitive tomato cultivars [11].

The relationship between infection, accumulation, and disease symptom expression of PSTVd has been reported in several previous studies. Co-inoculation with PSTVd and PSTVd-dsRNA decreased PSTVd accumulation, and the onset of symptoms was delayed and less severe compared with those of the control plants [35]. Moreover, infection and accumulation of PSTVd were suppressed in transgenic potato plants expressing dsRNA-specific ribonuclease pac1 protein, and these plants did not develop disease symptoms at different study temperatures: 25–32 (favorable for viroid replication and accumulation) and 20–28 °C (resembling actual field conditions) [36]. Similarly, transgenic potato plants expressing a hammerhead ribozyme targeting the minus-strand RNA of PSTVd-dsRNA inoculated with PSTVd did not develop disease symptoms, and viroid accumulation in the plant was decreased or at undetectable levels [37]. Alternatively, in transgenic tomato plants expressing hairpin (hp) RNA constructs derived from PSTVd sequences, symptom expression was correlated with PSTVd accumulation, and resistance was observed in plants accumulating high-level hpPSTVd and vd-sRNA [38]. These findings suggest that maintaining PSTVd accumulation at a low or an undetectable level leads to PSTVd tolerance or resistance.

Therefore, in this study, to evaluate PSTVd infection responses including PSTVd resistance and tolerance, which are expected to be applicable in tomato breeding, we inoculated cultivated and wild tomatoes and wild tomato relatives with intermediate and lethal strains of PSTVd. High PSTVd tolerance, accompanied by low PSTVd accumulation, was discovered in two wild relatives. We generated F1 hybrids by crossing sensitive wild tomatoes with tolerant wild tomato relatives and determined that the tolerance found in wild tomato relatives can be introduced into sensitive tomatoes by crossing. Our data will provide new insights into the mechanisms of PSTVd tolerance and disease symptom expression in tomato plants.

## 2. Results

### 2.1. Criteria for Assessing Sensitivity and Tolerance to PSTVd

**Figure 1:**
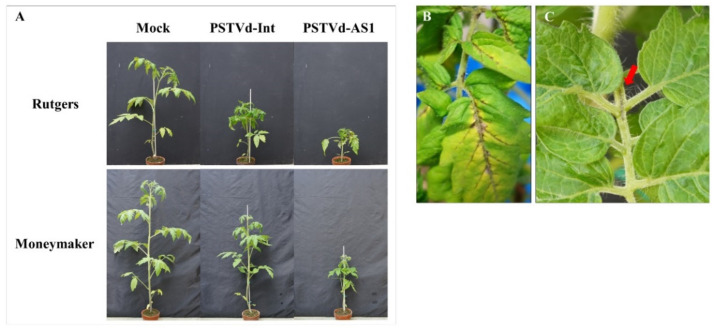
Symptoms in ‘Rutgers’ and ‘Moneymaker’ tomatoes infected with potato spindle tuber viroid (PSTVd). (**A**) At 3 weeks post-inoculation (wpi), PSTVd-infected ‘Rutgers’ tomatoes showed severe stunting and leaf curling. At 4 wpi, ‘Moneymaker’ tomatoes showed stunting during PSTVd infection, and mild leaf curling was observed only in PSTVd-AS1 infection. (**B,C**) Severe stem and vein necrosis, accompanied by yellowing or mild stem necrosis (indicated with a red arrow), was observed in ‘Rutgers’ and ‘Moneymaker’ tomatoes, respectively, after PSTVd-AS1 infection.

First, we confirmed PSTVd disease symptoms in ‘Rutgers’ and ‘Moneymaker’ tomatoes as criteria for assessing sensitivity and tolerance to PSTVd under our test conditions. In ‘Rutgers’ tomatoes, severe vein necrosis, accompanied by leaf yellowing, was observed during PSTVd-AS1 infection, in addition to stunting, leaf curling, and mild vein necrosis found in plants infected with PSTVd-Int (Figure 1). Meanwhile, ‘Moneymaker’ tomatoes, a PSTVd-tolerant cultivar, exhibited mild symptoms compared with ‘Rutgers’ tomatoes, and only stunting was observed during PSTVd-Int infection. However, PSTVd-AS1 infection induced severe stunting, leaf curling, size reduction of leaves, and mild stem necrosis even in PSTVd-tolerant ‘Moneymaker’ tomatoes (Figure 1). Using these symptoms as a baseline, the degree of sensitivity and tolerance and the various responses to PSTVd infection in the remaining tomato cultivars and wild tomato species were evaluated.

None of the host plants used in this study demonstrated sufficient PSTVd resistance to render systemic PSTVd infection impossible; however, these plants exhibited various responses, from asymptomatic to lethal, during PSTVd infection (Table 1 and Table 2).

### 2.2. The Relationship between Tomato Fruit Size and the Level of Tolerance to PSTVd

**Figure 2:**
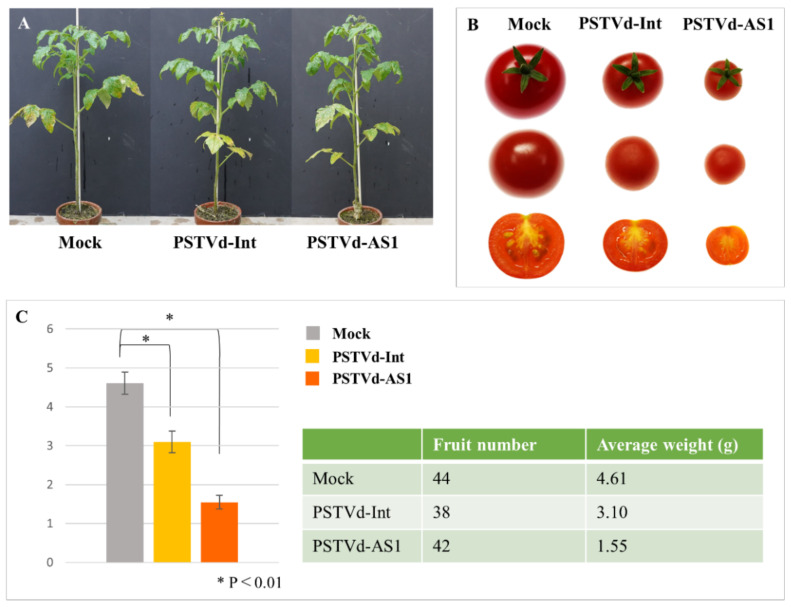
Symptoms of fruits in PSTVd-tolerant ‘Tiny-Tim’ tomatoes. (**A**) ‘Tiny-Tim’ tomatoes did not clearly show morphological symptoms in stems and leaves at 6 weeks post-inoculation. (**B**) The fruits from PSTVd-infected plants became significantly smaller in size and lost their surface luster; in particular, the fruits from PSTVd-AS1-infected plants did not form any seeds. (**C**) The average weight of fruits derived from PSTVd-infected plants was low compared with that from mock-inoculated, but there was no difference in total number of fruits. The significant difference in the fruit weight was confirmed by the Tukey–Kramer test.

In general, small-fruited tomatoes tend to be more tolerant to PSTVd than large- or medium-fruited tomatoes. Out of the tomato cultivars, the large-fruited cultivars were more sensitive but some medium- or small-fruited cultivars were less sensitive to PSTVd-AS1 infection (Table 1). For instance, ‘Tiny-Tim’ and ‘Micro-Tom’ tomatoes were more tolerant than ‘Moneymaker’ tomatoes to PSTVd, and no significant morphological symptoms were observed in the stems and leaves of the plants infected with both PSTVd strains up to 6 weeks post-inoculation (wpi) (Figure 2A and Figure S1A). Moreover, these PSTVd-infected plants were transplanted and grown to observe their fruits. The fruits from PSTVd-infected plants grew significantly smaller in size and lacked lustrous surfaces; the fruits from PSTVd-AS1-infected plants did not produce any seeds (Figure 2B,C and Figure S1B). Interestingly, the number of flower buds seemed to increase in PSTVd-infected plants, but the final number of fruits was almost the same as that in mock-inoculated plants (Figure 2C). Furthermore, the difference in pathogenicity between PSTVd strains was evident from the fruits’ symptoms (Figure 2B,C and Figure S1B). These results indicate that some symptoms may be observed in the fruits of small-fruited tomato cultivars, in which clear morphological symptoms are not observed during PSTVd infection.

### 2.3. Wild Tomatoes Contain Sensitive or Tolerant Accessions to PSTVd Infection

**Figure 3:**
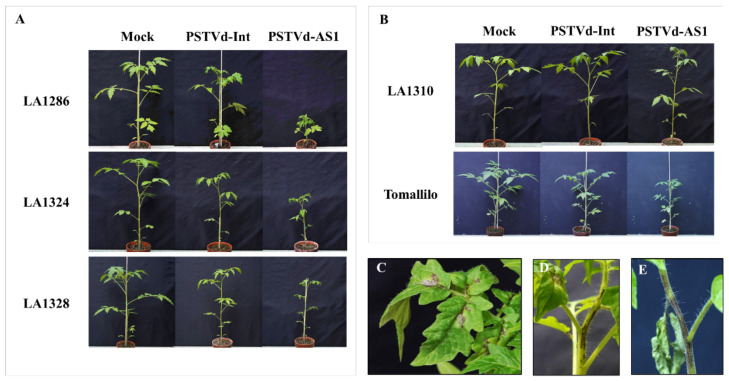
Wild tomatoes (*S. l. cerasiforme*) including PSTVd-sensitive or -tolerant accessions. Wild tomato accessions showed sensitivity or tolerance to PSTVd infection. (**A**) Three accessions of wild tomatoes were PSTVd-sensitive and showed severe stunting and leaf curling during PSTVd infection. (**B**) Two accessions of wild tomatoes were PSTVd-tolerant; LA1310 plants exhibited shortening of petiole length but no significant symptoms during PSTVd-AS1 infection. Stunting and mild leaf curling were observed only in ‘Tomallilo’ plants infected with PSTVd-AS1. (**C**,**D**) Severe vein and stem necroses were observed in PSTVd-sensitive wild tomato LA1286 during PSTVd-AS1 infection. (**E**) Highly severe stem necrosis in LA1324 plants resulted in withering.

Inoculation of wild tomato *S. l. cerasiforme* with PSTVd revealed that three accessions of *S. l. cerasiforme* (LA1286, LA1324, and LA1328) were sensitive to PSTVd, whereas two (LA1310 and the ‘Tomallilo’ cultivar) were tolerant to intermediate and lethal PSTVd strains (Figure 3A,B, Table 2). Of the PSTVd-sensitive accessions, LA1286 plants showed severe vein and stem necrosis as seen in ‘Rutgers’ tomatoes infected with PSTVd-AS1, and some LA1324 individuals inoculated with PSTVd-AS1 presented with hypersensitivity and died due to extremely severe stem necrosis (Figure 3C–E). In addition, symptoms commonly observed in tomato cultivars, such as stunting and leaf curling, were also observed in all PSTVd-sensitive accessions of wild tomatoes (Figure 3A). Meanwhile, of the PSTVd-tolerant accessions, LA1310 plants displayed a shortening of petiole lengths but no significant symptoms, and ‘Tomallilo’ plants only showed stunting and mild leaf curling when infected with PSTVd-AS1 (Figure 3B). Thus, it was shown that the wild tomato *S. l. cerasiforme*, the so-called cherry tomato, contained PSTVd-sensitive accessions that showed disease symptoms qualitatively similar to those in PSTVd-infected cultivated tomatoes, in addition to those exhibiting PSTVd tolerance, as previously reported [28].

### 2.4. Some Wild Tomato Relatives Exhibited PSTVd Tolerance Regardless of PSTVd Strain

**Figure 4:**
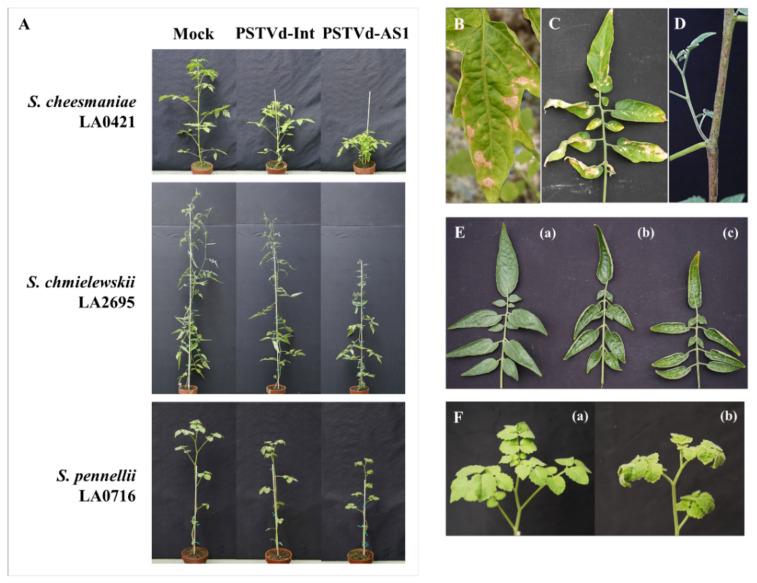
Wild tomato relatives showing characteristic symptoms during PSTVd infection. (**A**) In *S. cheesmaniae* LA0421, *S. chmielewskii* LA2695, and *S. pennellii* LA0716, clear symptoms including stunting and leaf miniaturization were observed after PSTVd infection. (**B**–**D**) Necrosis was observed in wild tomato relatives infected with PSTVd-AS1. (**B**) Leaf necrosis in *S. cheesmaniae* LA0421. (**C**) Leaf necrosis, accompanied by yellowing, and (**D**) stem necrosis in *S. chmielewskii* LA2695. (**E**) Leaf rolling was observed in PSTVd-infected *S. chmielewskii* LA2695 plants ((a), mock; (b), PSTVd-Int; (c), PSTVd-AS1). (**F**) *S. pennellii* LA0716 clearly exhibited leaf curling after PSTVd-AS1 infection ((a), mock; (b), PSTVd-AS1).

In ten wild tomato relatives (10 accessions), some disease symptoms were observed during PSTVd infection; for example, stunting occurred in all PSTVd-sensitive wild tomato relatives (Figure 4 and Figure S2, Table 2). Characteristic disease symptoms were observed in some wild tomato relatives. In *S. cheesmaniae* LA0421 and *S. chmielewskii* LA2695 plants, necrosis was observed on leaf tissue other than the veins, unlike in cultivated and wild tomatoes (Figure 4B–D), and leaf curling or rolling was observed only in *S. chmielewskii* LA2695 or *S. pennellii* LA0716 plants (Figure 4E,F). In addition to these wild tomato relatives, *S. arcanum* LA1031, *S. neorickii* LA0247, and *S. huaylasense* LA1358 were newly identified as symptomatic host plants in this assay (Figure S2, Table 2).

**Figure 5:**
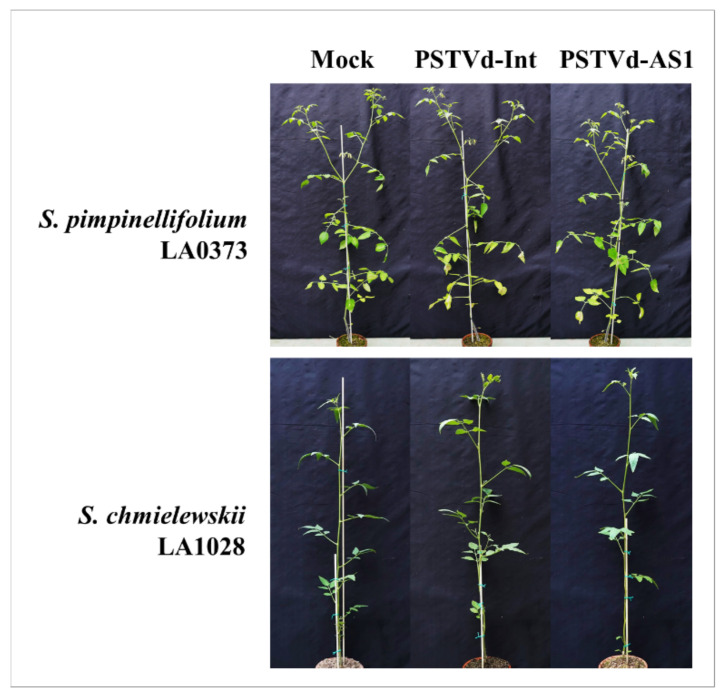
PSTVd tolerance in wild tomato relatives. *S. pimpinellifolium* LA0373 and *S. chmielewskii* LA1028 plants exhibited high PSTVd tolerance and did not show any disease symptoms at 6 weeks post-inoculation, even to PSTVd-AS1 infection.

In contrast, *S. pimpinellifolium* LA0373, *S. pimpinellifolium* LA0411, *S. galapagense* LA0317, and *S. chmielewskii* LA1028 plants displayed no significant symptoms and were tolerant to two distinct PSTVd strains (Figure 5 and Figure S3, Table 2). In particular, *S. pimpinellifolium* LA0373 and LA0411 and *S. chmielewskii* LA1028 plants were highly tolerant to PSTVd. These results indicate that wild tomato relatives present with various responses to PSTVd infection.

### 2.5. Low Accumulation of PSTVd in Wild Tomato Relatives Exhibiting High Tolerance to PSTVd

To obtain insight into the properties of PSTVd tolerance observed in some wild tomato relatives, such as *S. pimpinellifolium* LA0373 and *S. chmielewskii* LA1028, the accumulation of PSTVd in these accessions was analyzed by dot-blot hybridization.

**Figure 6:**
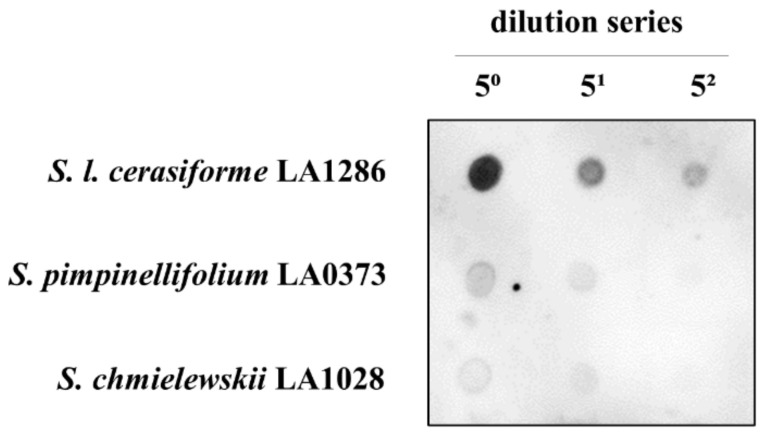
Low PSTVd accumulation in wild tomato relatives. Accumulation of PSTVd in wild tomato relatives was analyzed by dot-blot hybridization with a PSTVd-specific cRNA probe. Accumulation of PSTVd in *S. pimpinellifolium* LA0373 and *S. chmielewskii* LA1028 was lower than in a PSTVd-sensitive *S. l. cerasiforme* LA1286 at 3 weeks post-inoculation.

This assay revealed that the accumulation of PSTVd was lower in both of the wild tomato relatives than in the PSTVd-sensitive wild tomato, *S. l. cerasiforme* LA1286 (Figure 6), suggesting that the low accumulation of PSTVd is important for PSTVd tolerance in wild tomato relatives.

### 2.6. F1 Hybrids between a PSTVd-Sensitive Wild Tomato and PSTVd-Tolerant Wild Relative Were Tolerant to PSTVd

To understand the properties of PSTVd tolerance observed in wild tomato relatives in more detail, F1 hybrids were generated by crossing PSTVd-sensitive wild tomato *S. l. cerasiforme* LA1286 with *S. pimpinellifolium* LA0373 or *S. chmielewskii* LA1028. Subsequently, the F1 hybrids were inoculated with PSTVd-AS1.

**Figure 7:**
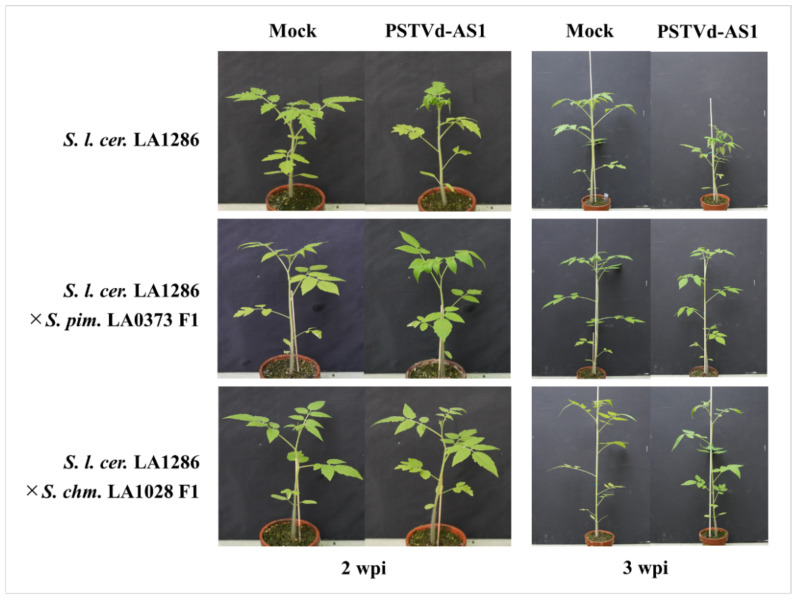
F1 hybrids between a PSTVd-sensitive wild tomato and PSTVd-tolerant wild relatives were tolerant to PSTVd. At 2 weeks post-inoculation (wpi), *S. l. cerasiforme* LA1286 showed severe leaf curling, whereas *S. l. cerasiforme* LA1286 × *S. pimpinellifolium* LA0373 F1 or *S. l. cerasiforme* LA1286 × *S. chmielewskii* LA1028 F1 showed very mild leaf curling or was asymptomatic. At 3 wpi, *S. l. cerasiforme* LA1286 exhibited stunting and vein necrosis, whereas *S. l. cerasiforme* LA1286 × *S. pimpinellifolium* LA0373 F1 recovered from mild symptoms and both F1 hybrids were asymptomatic even after lethal PSTVd-AS1 infection.

At 2 wpi, *S. l. cerasiforme* LA1286 plants showed severe leaf curling, whereas *S. l. cerasiforme* LA1286 × *S. pimpinellifolium* LA0373 F1 plants displayed very mild leaf curling on the leaves at the top. At 3 wpi, *S. l. cerasiforme* LA1286 plants exhibited clear stunting and vein necrosis, whereas *S. l. cerasiforme LA1286* × *S. pimpinellifolium* LA0373 F1 plants recovered and presented with no apparent symptoms compared with mock-inoculated plants (Figure 7). Alternatively, *S. l. cerasiforme* LA1286 × *S. chmielewskii* LA1028 F1 plants were asymptomatic at 2 and 3 wpi, despite lethal PSTVd strain infection (Figure 7).

These results indicate that the nature of symptom expression in PSTVd-sensitive wild tomatoes can be suppressed by that of tolerance in PSTVd-tolerant wild tomato relatives, even if infected with lethal PSTVd-AS1.

### 2.7. Low Accumulation of PSTVd Early in Infection in PSTVd-Tolerant F1 Hybrids

**Figure 8:**
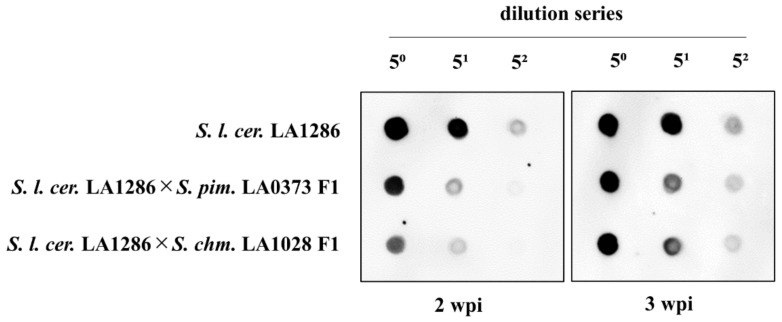
Low PSTVd accumulation in PSTVd-tolerant F1 wild tomatoes. Accumulation of PSTVd in wild tomato relatives was analyzed by dot-blot hybridization with a PSTVd-specific cRNA probe. At 2 weeks post-inoculation (wpi), accumulation of PSTVd decreased in F1 hybrids, especially in *S. l. cerasiforme* LA1286 × *S. chmielewskii* LA1028 F1 plants, compared with *S. l. cerasiforme* LA1286 plants. Afterward, at 3 wpi, the accumulation in F1 hybrids increased to an indistinguishable level compared with *S. l. cerasiforme* LA1286 plants at 5⁰ dilution.

Subsequently, the accumulation of PSTVd was analyzed in PSTVd-sensitive wild tomato *S. l. cerasiforme* LA1286 and PSTVd-tolerant F1 hybrids. At 2 wpi, the accumulation of PSTVd was lower in F1 hybrids, especially in *S. l. cerasiforme* LA1286 × *S. chmielewskii* LA1028 F1 plants, compared with *S. l. cerasiforme* LA1286 plants. At 3 wpi, the accumulation in F1 hybrids increased to an indistinguishable level compared with *S. l. cerasiforme* LA1286 plants at 5⁰ dilution (Figure 8). These results indicate that the severity of disease symptoms positively correlates with the early accumulation of PSTVd and suggest that suppression of PSTVd accumulation, particularly early in infection, is important to exhibit PSTVd tolerance.

### 2.8. Nicotiana Occidentalis Exhibits Severe Symptoms during Infection of Lethal PSTVd Strain

In our inoculation assay of tomato cultivars and wild tomato species, there were cases where expression of clear symptoms was observed only after inoculation with a large amount of infectious PSTVd-AS1 RNA (5 µg/plant). Therefore, host plants, even those reported to be asymptomatic or to exhibit only mild symptoms during PSTVd infection in previous reports, may develop disease symptoms after PSTVd-AS1 inoculation at high concentrations. For this reason, we also examined responses to two PSTVd strains in five different species of the family Solanaceae under the same conditions as the inoculation method utilized for tomato cultivars and wild tomato species.

**Figure 9:**
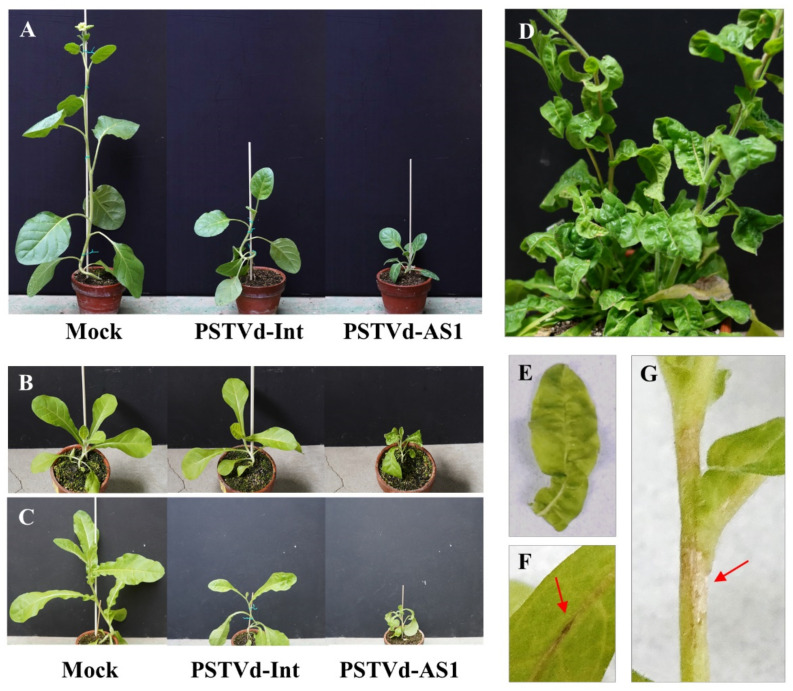
Symptoms in Nicotiana plants infected with PSTVd. (**A**) At 8 weeks post-inoculation (wpi), stunting, leaf miniaturization, and poor growth were observed in PSTVd-infected *N. rustica* plants and were more severe during PSTVd-AS1 infection. (**B**) At 3 wpi, *N. occidentalis* plants inoculated with 5 μg RNA per plant clearly exhibited disease symptoms only after inoculation with PSTVd-AS1. (**C**) At 3 wpi, *N. occidentalis* plants inoculated with 10 μg RNA per plant exhibited stunting even by inoculation with PSTVd-Int. (**D**–**G**) *N. occidetalis* plants infected with PSTVd-AS1 exhibited disease symptoms such as leaf curling, rugose, and vein/stem necrosis in addition to stunting. (**D**) Rugose and leaf curling at 7 wpi. (**E**) Leaf rugose at 3 wpi. (**F**) Vein necrosis at 3 wpi (indicated with a red arrow). (**G**) Stem necrosis at 4 wpi (indicated with a red arrow).

As expected, potato (*Solanum tuberosum*) plants, a symptomatic host, inoculated with PSTVd-AS1 exhibited more severe symptoms than those with PSTVd-Int (Table 3). An eggplant cultivar ‘Black Beauty’ (*Solanum melongena*) was reported as a symptomatic host [39]; however, in our assay, two eggplant cultivars ‘Kurobe’ and ‘Kurowashi’ did not exhibit any symptoms regardless of the PSTVd strain. In addition, two cultivars of capsicum (*Capsicum annuum*) plants, an asymptomatic host of PSTVd in the previous report [40], also did not exhibit any symptoms regardless of the PSTVd strain. All inoculated capsicums and eggplants were confirmed to be infected with PSTVd by RT-PCR. On the other hand, *Nicotiana rustica* plants exhibited apparent stunting, leaf miniaturization, and poor growth during PSTVd infection, inconsistent with a previous report [40] (Figure 9A, Table 3). Symptoms of stunting in *Nicotiana* plants have been reported only in *N. benthamiana* infected with PSTVd-AS1 or PSTVd-Nb [11,41]. No reports were found about an inoculation assay of *N. occidentalis* with PSTVd as far as we searched. Interestingly, *N. occidentalis* plants exhibited disease symptoms, such as stunting, leaf curling, rugose, and vein/stem necrosis, similar to those observed in PSTVd-infected tomatoes, only after PSTVd-AS1 infection (Figure 9B,D–G, Table 3). It should be noted that stunting in PSTVd-Int-infected *N. occidentalis* was observed only when the plants were inoculated with an excessive amount of PSTVd-Int RNA (10 µg/plant) and were grown under greenhouse conditions suitable for disease onset (Figure 9C, Table 3).

These results indicate that some asymptomatic host plants may be capable of developing disease symptoms after inoculation with high concentrations of a PSTVd inoculum or inoculation with a lethal PSTVd isolate. Symptoms of stunting in *Nicotiana* plants have been reported only in *N. benthamiana* infected with PSTVd-AS1 or PSTVd-Nb [11,41]. For the first time in this study, *N. rustica* and *N. occidentalis* were shown to develop disease symptoms during PSTVd infection. At present, *N. occidentalis* is considered the only *Nicotiana* plant that exhibits obvious disease symptoms such as rugose, leaf curling, and vein/stem necrosis due to PSTVd infection.

## 3. Discussion

To date, effective control methods for viroid diseases have not been established; therefore, the use of tolerant or resistant cultivars to viroid infection is considered the most effective countermeasure against viroid diseases. However, in most crops, except for chrysanthemums, which have been reported to show CSVd resistance in some cultivars, viroid-resistant cultivars or traits available in breeding have not been reported. Similarly, tomato, an agriculturally important crop, is a pathogenic host of many pospiviroids, but no resistance has been reported. In addition, although wild tomato relatives contain many species that can be crossed with cultivated tomatoes, responses to PSTVd infection have been clarified only in some species. Therefore, this study was conducted to clarify the responses of a wide range of cultivated and wild tomatoes and wild tomato relatives to PSTVd infection and to examine genetic resources of PSTVd resistance that could be introduced into susceptible cultivated tomatoes by crossing. No tomato cultivars or wild tomato species exhibiting PSTVd resistance capable of rendering systemic infection of PSTVd impossible were found, but the tested plants exhibited various responses, including high tolerance to PSTVd infection (Table 1 and Table 2).

Cultivated tomatoes are derived from *S. l. cerasiforme* (wild tomatoes) and *S. pimpinellifolium* (the putative closest wild tomato relative) [21], which bear small fruits. Moreover, in the process of domestication and subsequent breeding, tomatoes accumulated mutations in the genome that increased their fruit size, resulting in the larger fruits of the currently cultivated tomatoes [18,22,23,24]. In this study, the small-fruited tomato cultivars tended to have a higher PSTVd tolerance than the large- and medium-fruited tomato cultivars (Figure 1 and Figure 2, Table 1). These findings suggest that there is a link between the increase in fruit size associated with tomato domestication/breeding and the high sensitivity to PSTVd because ancestor wild species of cultivated tomatoes are asymptomatic during PSTVd infection [28]. Our inoculation assay revealed that *S. l. cerasiforme* included PSTVd-sensitive accessions in addition to those exhibiting PSTVd tolerance as previously reported [28] (Figure 3), and that *S. pimpinellifolium* did not develop any disease symptoms [28] (Figure 5). These results suggest that PSTVd sensitivity in tomatoes is derived from a group of PSTVd-sensitive *S. l. cerasiforme* because this species is a complex mixture of domesticated (close to cultivated tomatoes) and wild (close to *S. pimpinellifolium*) groups [22]. Our data on necrosis symptoms support the above hypothesis regarding the origin of PSTVd sensitivity. Necrosis was observed in some wild tomatoes and wild tomato relatives, as well as some tomato cultivars; the symptoms observed in cultivated and wild tomatoes infected with PSTVd appeared on the veins and stems, whereas necrosis was observed on the stems and leaf tissues other than the veins in *S. cheesmaniae* LA0421 and *S. chmielewskii* LA2695 (Figure 1B,C, Figure 3C–E, Figure 4B–D, Table 1 and Table 2). Considering these findings, PSTVd-sensitive or -tolerant tomato cultivars may have been domesticated/bred from the PSTVd-sensitive (close to cultivated tomatoes) or -tolerant (close to *S. pimpinellifolium*) wild tomato, respectively.

On the other hand, this possibility inadequately explains the high PSTVd sensitivity in large-sized tomato cultivars because the fruits of wild tomatoes are larger than those of *S. pimpinellifolium*, but clearly smaller than those of large-fruited tomato cultivars. In this study, stunting was generally observed in PSTVd-sensitive tomato cultivars, and the fruits of ‘Tiny-Tim’ and ‘Micro-Tom’ tomatoes showed disease symptoms, such as miniaturization, sterility, and loss of luster (Figure 1 and Figure 2B,C, Table 1). Stunting and fruit miniaturization were also observed in tomato planta macho viroid- or Mexican papita viroid-infected ‘Rutgers’ tomatoes in which the expression of gibberellin biosynthesis genes *GA20ox1* and *GA7ox*, and *GAI* (gibberellic acid insensitive) was suppressed [42]. The expression of these genes was also suppressed in PSTVd-infected tomato plants [17], and suppression of the StTCP23 transcription factor, which activates gibberellin metabolism, caused by PSTVd infection in potato plants, was associated with disease symptom expression [9]. These findings suggest that the gibberellin metabolism pathway fluctuation is associated with disease symptom expression in PSTVd-infected host plants. Gibberellin is also involved in the fruit development of tomatoes by regulating fruit set and cell expansion after flowering [43,44]. It has been suggested that the expression of the *YABBY-like* transcription factor gene, which regulates gibberellin synthesis by feedback control in rice, was suppressed in modern cultivated tomatoes due to the large insertion into the first intron region of the gene so that the number of locules was increased and the size of fruits was enlarged [24,45]. Considering these reports, there may be a relationship between tomato fruit size and symptom expression (or PSTVd sensitivity) via gibberellin metabolism. Further research is needed to confirm this possibility.

None of the host plants demonstrated PSTVd resistance in this study, but some wild tomato relatives showed high tolerance to PSTVd. Little research has been conducted on tolerance to viroids, and the mechanism and its properties have not been elucidated. In previous reports, F1 hybrids obtained by crossing sensitive and tolerant tomato cultivars had the dominant trait of developing disease symptoms [11]. In our study, F1 hybrids generated by crossing between symptomatic *S. l. cerasiforme* LA1286 and asymptomatic *S. pimpinellifolium* LA0373 or *S. chmielewskii* LA1028 exhibited tolerance even to lethal PSTVd-AS1 (Figure 7), indicating that PSTVd tolerance is a dominant trait and can be introduced into PSTVd-sensitive tomatoes by crossing. This is the first report demonstrating that PSTVd tolerance can be introduced into PSTVd-sensitive tomatoes by general crossing, which does not use transformation techniques. Since *S. pimpinellifolium* and *S. chmielewskii* are wild tomato relatives that can be crossed with not only wild tomatoes (*S. l. cerasiforme*) but also cultivated tomatoes, this tolerance to PSTVd is a high-value trait in breeding.

Inoculation with different PSTVd strains in a wide range of host plants is useful in comprehensively understanding the relationship between host and viroid, in the viroid pathogenesis. Our data show that pathogenicity differences between PSTVd strains were evident in PSTVd-sensitive host plants, and not in highly tolerant or asymptomatic hosts (Table 1, Table 2 and Table 3), thus suggesting that the role of pathogenicity determinants of the viroid depends on the host plant and that the severity of symptoms in the plant is determined by the interaction between viroid pathogenicity determinants and host factors related to symptom expression. In this study, during PSTVd-AS1 infection, PSTVd-tolerant wild tomato relatives had a lower accumulation of PSTVd than PSTVd-sensitive wild tomatoes and the severity of disease symptoms in their F1 hybrids demonstrated a positive correlation with early accumulation (Figure 5, Figure 6, Figure 7 and Figure 8). In a previous study, suppression of PSTVd accumulation resulted in suppression of symptom expression or reduced the degree of disease symptoms [35,37,38]. These findings suggest that *S. pimpinellifolium* LA0373 and *S. chmielewskii* LA1028 contain factors that affect PSTVd accumulation, which is one of the determinants of disease symptom severity.

Regarding the low accumulation of PSTVd in wild tomato relatives, it is possible that PSTVd replication and intercellular/systemic transfer were suppressed or that the accumulated PSTVd was degraded by the anti-viroid defense mechanism, including RNA silencing. In previous reports, suppression of DCL2 and DCL4 in tolerant ‘Moneymaker’ tomatoes increased the accumulation and sensitivity during PSTVd, PVX, or PVY infection [46,47]. It has also been reported that the combined activity of DCL2 and DCL3 in *N. benthamiana* is important for suppressing the disease symptom expression of PSTVd [48]. These findings suggest that the degradation mechanisms of viroids, including RNA silencing, are involved in suppressing PSTVd accumulation, thereby exhibiting PSTVd tolerance in *S. pimpinellifolium* LA0373 and *S. chmielewskii* LA1028.

Suppressed accumulation of PSTVd early during infection in wild relatives and their F1 hybrids may also be associated with pathogen-associated molecular patterns (PAMPs)-triggered immunity (PTI), which is generally considered as the defense response of plants in early infection of pathogens. For instance, the earliest signaling events in PTI responses include the influx of Ca^2+^, production of reactive oxygen species, and activation of mitogen-activated protein kinase (MAPK) cascades [49]. In PSTVd infection, induction of PTI responses was suggested from comprehensive transcriptome analyses in ‘Heinz 1706’ tomatoes, and MAPK3 was induced at both transcriptional and translational levels [50]; although the involvement of PTI in defense responses against viroids, including RNA silencing, has been suggested in a previous study [51], no studies have been conducted to prove that hypothesis. At 14 dpi, PSTVd accumulation was lower in F1 hybrids than in *S. l. cerasiforme* LA1286 but increased to an indistinguishable level at 5⁰ dilution at 21 dpi (Figure 8). A similar phenomenon was observed in PSTVd-tolerant ‘Moneymaker’ tomatoes whose symptoms were exacerbated by the downregulation of DCL2 and DCL4 [46]. Considering these findings, suppression of PSTVd accumulation in early infection may be important to exhibit PSTVd tolerance. The plants may probably gain time to activate their anti-viroid defense by suppressing the initial accumulation, resulting in tolerance to PSTVd.

Alternatively, the severity of disease symptoms and accumulation of PSTVd is likely to be associated with the number of genes that fluctuate due to PSTVd infection. In general, mild strains of PSTVd accumulate less than highly pathogenic strains [11,12,52]. Additionally, microarray analyses in ‘Rutgers’ tomatoes infected with mild or severe strains of PSTVd revealed that 3037 differentially expressed genes were observed in severe strain infection, whereas less than one third of those were found in mild strain infection [53], suggesting that fluctuations in the expression levels of many genes disrupt the normal metabolism of plants and cause disease symptoms. In this study, low accumulation of PSTVd in F1 plants correlated with the moderation of disease symptom severity (Figure 7 and Figure 8). These findings suggest that the suppression of PSTVd accumulation by crossing with PSTVd-tolerant wild tomato relatives results in a decrease in the number of fluctuating host genes and direct or indirect suppression of disease symptom expression. The mechanism of PSTVd tolerance and suppression of PSTVd accumulation in wild relatives will be further analyzed in the future.

In conclusion, this study demonstrated that wild tomato relatives (*S. cheesmaniae*, *S. galapagense*, *S. chmielewskii*, *S. arcanum*, *S. neorickii*, *S. huaylasense*, and *S. pennellii*) whose response to PSTVd infection was unknown exhibit various responses during PSTVd infection and that many of them, *S. l. cerasiforme*, *N. rustica*, and *N. occidentalis*, are symptomatic hosts of PSTVd. At present, *N. occidentalis* is considered the only *Nicotiana* plant that exhibits obvious disease symptoms such as rugose, leaf curling, and vein/stem necrosis due to PSTVd infection. Furthermore, although no host plants exhibited PSTVd resistance, we found highly PSTVd-tolerant wild tomato relatives that displayed almost no symptoms during infection with intermediate and lethal strains of PSTVd. This PSTVd tolerance in *S. pimpinellifolium* LA0373 and *S. chmielewskii* LA1028 is a dominant trait that can be utilized for tomato breeding. This is the first study to produce F1 hybrid tomatoes showing PSTVd tolerance by crossing, along with the low accumulation of PSTVd in early infection; however, the detailed mechanism is unknown. In the future, we plan to reveal the mechanism of PSTVd tolerance and suppression of PSTVd accumulation in wild relatives. Our data will provide new insights into disease tolerance and the mechanism of symptom expression.

## 4. Materials and Methods

### 4.1. Plant Materials

Tomatoes (1 species, 13 cultivars), wild tomatoes (1 species, 5 accessions), wild tomato relatives (12 species, 14 accessions), and another five species of the family Solanaceae (including the genus *Capsicum*, *Nicotiana*, and *Solanum*) were used for the inoculation assay with two PSTVd strains in order to evaluate responses to PSTVd infection. Open pollinated (OP) seeds of the following tomato cultivars were purchased from seed companies in Japan: ‘Berner Rose’ (Tsurusin Seed Co., Ltd, Nagano, Japan), ‘Pondelosa’ and ‘Sekai-ichi’ (Noguchi Seeds Co., Saitama, Japan), and ‘Lemon-tomato’ and ‘San Marzano’ (SNC Sapporo Nouen Co., Ltd., Hokkaido, Japan). OP seeds of tomato cultivars ‘Moneymaker’, ‘Newskij’, and ‘Rutgers’ were collected in our laboratory. In addition, OP seeds of the following tomato cultivars were kindly provided by the following institutions: ‘Micro-Tom’ by the Tomato Genetics Resource Center (TGRC), and ‘Sugar’ and ‘Tiny-Tim’ by the National Plant Germplasm System (NPGS). F1 hybrid seeds of tomato cultivars ‘Momotaro’ and ‘Chika’ were produced by the seed company of Japan (Takii & Co., Ltd., Kyoto, Japan). All seeds of wild tomato species were kindly provided by the TGRC or the NPGS. OP seeds of *N. occidentalis* and *N. rustica* were collected in our laboratory. OP seeds of capsicum cultivars ‘Botan-kosho’ and ‘Takano-tsume’ were purchased from seed companies (Tsurusin Seed and Sakata Seed Corporation, Kanagawa, Japan, respectively). F1 hybrid seeds of eggplant cultivars ‘Kurobe’ and ‘Kurowashi’ were produced by seed companies of Japan (Watanabe Seed Co., Ltd., Miyagi, Japan, and Takii & Co., Ltd., respectively).

*S. l. cerasiforme* LA1286 was crossed as a seed parent with a wild tomato relative, *S. pimpinellifolium* LA0373 or *S. chmielewskii* LA1028, as a pollen parent, and F1 hybrid seeds were produced in this study.

Seeds of wild tomato species were sown on filter paper after pretreatment with 2.7% hypochlorous acid for 30 or 60 min to improve the germination rate. After germination, seedlings were planted in potting soil and grown in a greenhouse with controlled temperature at 24 (night)–28 °C (day), with a day length of about 16 h with light provided by the sun and a sunlight lamp. Once the plants unfolded two true leaves (approximately 10–12 days after transplantation), they were inoculated with PSTVd and grown under similar growth conditions for at least 6 wpi. An inoculation assay was repeated at three different times, and at least three individual plants were used per inoculum in every assay.

### 4.2. In Vitro Transcription of PSTVd RNA and Mechanical Inoculation

Infectious PSTVd transcripts of intermediate (isolate PSTVd-Int, Accession No. M16826) and lethal (isolate PSTVd-AS1, Accession No. AY518939) strains were used for the inoculation assay. After in vitro transcription with T7 RNA polymerase (Invitrogen, Carlsbad, CA, USA), a template plasmid was digested with DNase I (RT Grade) (Nippon Gene, Toyama, Japan), and transcribed RNA was collected by ethanol precipitation using ammonium acetate to remove substrate nucleotides, before suspension in RNA storage solution (Ambion). The concentration and the size of transcripts were confirmed using a NanoDrop-1000 (Thermo Fisher Scientific, Waltham, MA, USA) and denaturing agarose gel electrophoresis containing 2.2 M formaldehyde. PSTVd inoculums were adjusted to include 0.1 M Tris-HCl (pH 7.5), 0.01 M EDTA (pH 7.5), and 0.33 µg/µL bentonite at the final concentration. When evaluating PSTVd infection response (Section 2.1, Section 2.2, Section 2.3, Section 2.4, Section 2.5, Section 2.6, Section 2.7 and Section 2.8) or analyzing PSTVd tolerance (Section 2.5, Section 2.6 and Section 2.7), 5 or 2 µg infectious PSTVd RNA per individual plant was used for inoculation, respectively. Exceptionally, *N. occidentalis* was inoculated with 5 or 10 µg RNA per plant (Section 2.8). For mechanical inoculation, an aliquot of inoculum (2–4 µL containing infectious PSTVd RNA) was placed on the second true leaf from the top of individual plants (2−4 true leaf stage) dusted with carborundum (600 mesh) and gently rubbed ten times against the leaf using a finger covered in a finger cot.

### 4.3. Isolation of Nucleic Acid and Confirmation of PSTVd Infection by RT-PCR

A total of five leaf disks (6 mm in diameter) were collected at 4 wpi from individual PSTVd-inoculated plants for nucleic acid extraction. Total nucleic acids were extracted by a rapid method using potassium ethyl xanthogenate, as described previously [54], with some modifications (Hataya, submitted). The nucleic acid suspension was used for subsequent confirmation of PSTVd infection by RT-PCR.

In the RT reaction, cDNA was synthesized from 1 µL of the nucleic acid suspension using random hexamer primers (TaKaRa Bio Inc., Shiga, Japan) and ReverTra Ace (Toyobo, Osaka, Japan) and used as a template for PCR to amplify part of the PSTVd sequence. PCR was performed with Hot start *Taq* DNA polymerase (New England Biolabs, Ipswich, MA, USA) and a primer set, PSTV-7P and PSTV-7M [55]. The PCR products were fractionated in 1.5% agarose gel containing 0.5×Tris-acetate-EDTA buffer, stained with ethidium bromide solution (0.5 µg/mL), and visualized under ultraviolet (UV) irradiation at 312 nm.

### 4.4. Extraction of Total RNA and Detection of PSTVd by Dot-Blot Hybridization

Total RNA was extracted from the second true leaf from the top of individual plants inoculated with PSTVd-AS1 using RNAiso Plus (TaKaRa Bio Inc.) according to the manufacture’s protocol. Total RNA samples were used for dot-blot hybridization to detect PSTVd. A 5-fold dilution series from total RNA of 62.5 (in the case of wild relatives) or 31.25 ng (in the case of F1 hybrids) was prepared, spotted onto the Hybond-N membrane (GE Healthcare Life Sciences, Piscataway, NJ, USA), and crosslinked with UV irradiation. Hybridization was conducted with a digoxigenin-labeled cRNA probe for PSTVd [51,54]. Hybridized signals were visualized using ImageQuant LAS4000 (Fujifilm, Tokyo, Japan).

## Figures and Tables

**Table 1:** Symptoms of tomato cultivars infected with intermediate and lethal strains of PSTVd.

Cultivar	Fruit Size	Severity and Type of Disease Symptoms	
		PSTVd-Int (Intermediate)	PSTVd-AS1 (Lethal)
Berner Rose	Large	++ (S, Lc, Lm)	+++++ (S, Lc, Lm, Ru)
Momotaro (F1)	Large	+++ (S, Lc)	+++++ (S, Lc, Lm, Y, Ru)
Pondelosa	Large	++ (S, Lc)	+++++ (S, Lc, Lm, Y, Ru)
Sekai-ichi	Large	+++ (S, Lc, Lm)	+++++ (S, Lc, Lm, Y, Ru)
Lemon-tomato *^1^	Medium	+ (S)	++ (S, Lm)
Moneymaker	Medium	++ (S, Lc)	++++ (S, Lc, Lm, Sn)
Newskij	Medium	+ (Lc, Fm)	++++ (S, Lc, Lm, Y, Vn, Fm, Pcf, Lf, Ste)
Rutgers	Medium	++++ (S, Lc, Lm, Sn, Ru)	++++++ (S, Lc, Lm, Y, Ru, Vn, Sn)
San Marzano	Medium	++ (S, Lc)	+++++ (S, Lc, Lm, Ru, Sn)
Chika (F1)	Small	+ (S)	++ (S)
Micro-Tom	Small	+ (Fm, Pcf, Lf)	++ (Lm, Fm, Pcf, Lf, Ste)
Sugar *^1^	Small	−	+ (S, Lm)
Tiny-Tim	Small	+ (Fm, Pcf, Lf) *^2,3^	++ (Lm, Fm, Pcf, Lf, Ste) *^2,3^

The symbols “+” and “−” indicate symptomatic and asymptomatic, respectively. The higher the number of “+”, the more severe the symptoms of PSTVd. PSTVd-inoculated plants were observed until at least 6 weeks post-inoculation. Letters in parentheses mean the following symptoms: S, stunting; Lc, leaf curling (downward); Lm, leaf miniaturization; Ru, rugose; Sn, stem necrosis; Vn, vein necrosis; Y, yellowing; Fm, fruit miniaturization; Pcf, poor coloring fruit; Lf, lusterless fruit; Ste, sterility. *1. Growth of healthy or mock-inoculated plants tended to be suppressed under the conditions of our test. *2. Slightly poor growth was observed in PSTVd-infected plants. *3. The number of flower buds increased in PSTVd-infected plants.

**Table 2:** Symptoms of wild tomatoes and wild tomato relatives infected with intermediate and lethal strains of PSTVd.

Species	Cultivar /Accession	Severity and Type of Disease Symptoms	
		PSTVd-Int (Intermediate)	PSTVd-AS1 (Lethal)
*Solanum lycopersicum* var. *cerasiforme*	LA1286	+++ (S, Lc, Lm, Ru, Vn)	+++++ (S, Lc, Lm, Ru, Vn, Sn)
*Solanum lycopersicum* var. *cerasiforme*	LA1310	−	+ (Lm) *^1^
*Solanum lycopersicum* var. *cerasiforme*	LA1324	++ (S, Lc, Lm)	+++++ (S, Lc, Lm, Ru, Vn, Sn, W) *^2^
*Solanum lycopersicum* var. *cerasiforme*	LA1328	+ (S, Lm)	+++ (S, Lc, Lm, Vn)
*Solanum lycopersicum* var. *cerasiforme*	Tomallilo	+ (S, Lm) *^3^	++ (S, Lc, Lm) *^3^
*Solanum pimpinellifolium*	LA0373	−	− *^4^
*Solanum pimpinellifolium*	LA0411	−	− *^4^
*Solanum cheesmaniae*	LA0421	+++ (S, Lm, Ln)	++++ (S, Lr, Lm, Ln, Ro)
*Solanum galapagense*	LA0317	− *^5^	− *^5^
*Solanum chmielewskii*	LA1028	−	−
*Solanum chmielewskii*	LA2695	++ (S, Lr, Lm, Ln)	++++ (S, Lr, Lm, Ln, Sn, Y)
*Solanum arcanum*	LA1031	+ (S) *^6^	++ (S) *^6^
*Solanum neorickii*	LA0247	++ (S, Lm)	+++ (S, Lm)
*Solanum hyaylasense* *^7^	LA1358	+ (S)	+ (S)
*Solanum pervianum*	LA0111	++ (S, Lm, Ro)	+++ (S, Lm, Ro)
*Solanum corneliomulleri*	LA0103	+ (S) *^8^	++ (S, Ro) *^8^
*Solanum chilense*	LA1938	− *^9^	+ (S, Ro) *^9^
*Solanum habrochaites*	LA0361	+ (S) *^10^	++ (S, Ro) *^10^
*Solanum pennellii*	LA0716	++ (S, Lc, Ru)	+++ (S, Lc, Lm, Ru)

The symbols “+” and “−” indicate symptomatic and asymptomatic, respectively. The higher the number of “+”, the more severe the symptoms of PSTVd. PSTVd-inoculated plants were observed until at least 6 weeks post-inoculation (wpi). Letters in parentheses mean the following symptoms: S, stunting; Lc, leaf curling (downward); Lr, leaf rolling (upward); Lm, leaf miniaturization; Ln, leaf necrosis; Ru, rugose; Sn, stem necrosis; Vn, vein necrosis; Y, yellowing; Ro, rosette; W, withering. *1. Under the best conditions for disease onset, poor growth was observed in plants infected with PSTVd-AS1. *2. Some of the plants infected with PSTVd-AS1 died due to highly severe vein necrosis. *3. After 4 wpi, PSTVd-infected plants recovered. *4. Poor growth was observed in plants infected with PSTVd-AS1 when grown for more than 12 weeks. *5. The number of leaves and branches was reduced in PSTVd-infected plants. *6. Disease symptoms varied in severity between inoculated plants. *7. Growth of the healthy or mock-inoculated plants tended to be suppressed under our test conditions. *8. Stunting began to be observed in PSTVd-infected plants at 4 wpi and became apparent at 7 wpi. *9. Due to large differences in germination and growth, it was impossible to take biological and technical replicates. *10. PSTVd-infected plants were stunted after 6 wpi.

**Table 3:** Symptoms of five Solanaceae species infected with intermediate and lethal strains of PSTVd.

Species	Cultivar	Severity and Type of Disease Symptoms	
		PSTVd-Int (Intermediate)	PSTVd-AS1 (Lethal)
*Capsicum annuum*	Botan-kosho	−	−
*Capsicum annuum*	Takano-tsume	−	−
*Nicotiana occidentalis*		− *^1^	+++++ (S, Lc, Lm, Ru, Vn, Sn)
*Nicotiana rustica*		++ (S, Lm) *^2^	+++ (S, Lm) *^2^
*Solanum melongena*	Kurobe (F1)	−	−
*Solanum melongena*	Kurowashi (F1)	−	−
*Solanum tuberosum*	Danshaku-imo	++++ (S, Lm, Ro)	++++++ (S, Lm, Ru, Y, Ro, W)

The symbols “+” and “−” indicate symptomatic and asymptomatic, respectively. The higher the number of “+”, the more severe the symptom of PSTVd. PSTVd-inoculated plants were observed until at least 6 weeks post-inoculation. Letters in parentheses mean the following symptoms: S, stunting; Lc, leaf curling (downward); Lm, leaf miniaturization; Ru, rugose; Sn, stem necrosis; Vn, vein necrosis; Y, yellowing; Ro, rosette; W, withering. *1. PSTVd-Int-infected plants were stunted when they were inoculated with an excessive amount of PSTVd RNA (10 μg/plant). *2. Poor growth was observed in PSTVd-infected plants.

## Data Availability

The data presented in this study are available within the article and its Supplementary Materials.

## Supplementary Materials

The following are available online at https://www.mdpi.com/2223-7747/10/3/575/s1, Figure S1: Symptoms in fruits of PSTVd-tolerant ‘Micro-Tom’ tomatoes. ‘Micro-Tom’ tomatoes showed clear disease symptoms only in fruits derived from PSTVd-infected plants. (**A**) ‘Micro-Tom’ tomatoes exhibited PSTVd tolerance to some extent, and did not clearly show morphological symptoms in stems and leaves at 6 weeks post-inoculation. (**B**) The fruits from PSTVd-infected plants became significantly smaller in size and lost their surface luster; the fruits from PSTVd-AS1-infected plants did not form any seeds. Differences in pathogenicity between PSTVd strains were remarkable with regard to fruit symptoms; Figure S2: PSTVd-sensitive wild tomato relatives. In addition to the three species described in Figure 4, *S. arcunum* LA1031, *S. neorickii* LA0247, and *S. huaylasense* LA1358 were symptomatic during PSTVd infection. *S. arcanum* LA1031 plants exhibited stunting at 6 weeks post-inoculation (wpi). *S. neorickii* LA0247 showed stunt and leaf miniaturization at 6 wpi. *S. huaylasense* LA1358 exhibited stunting at 4 wpi, whereas plant growth tended to be suppressed under our test conditions. Differences in pathogenicity between PSTVd strains were remarkable in *S. arcanum* LA1031 and *S. neorickii* LA0247 plants; Figure S3: PSTVd-tolerant wild tomato relatives. In addition to the three species described in Figure 5, *S. pimpinellifolium* LA 0411 and *S. galapagense* LA0317 exhibited PSTVd tolerance regardless of PSTVd strain. *S. pimpinellifolium* LA0411 was asymptomatic during PSTVd infection at 6 weeks post-inoculation, similar to *S. pimpinellifolium* LA0373. *S. galapagense* LA0317 exhibited no obvious symptoms, except for poor growth, such as decreased number of branches and leaves.

## References

[1] Di Serio F., Li S.-F., Pallás V., Owens R.A., Randles J.W., Sano T., Verhoeven J.T., Vidalakis G., Flores R., Hadidi A., Flores R., Randles J.W., Palukaitis P. (2017). Viroid Taxonomy. Viroids and Satellites.

[2] Di Serio F., Flores R., Verhoeven J.T.J., Li S.-F., Pallás V., Randles J.W., Sano T., Vidalakis G., Owens R.A. (2014). Current status of viroid taxonomy. Arch. Virol..

[3] Daròs J.A., Flores R. (2004). *Arabidopsis thaliana* has the enzymatic machinery for replicating representative viroid species of the family Pospiviroidae. Proc. Natl. Acad. Sci. USA.

[4] Flores R., Hernández C., de Alba A.E.M., Daròs J.A., Di Serio F. (2005). Viroids and viroid–host interactions. Annu. Rev. Phytopathol..

[5] Kovalskaya N., Hammond R.W. (2014). Molecular biology of viroid-host interactions and disease control strategies. Plant Sci..

[6] Navarro B., Gisel A., Rodio M.E., Delgado S., Flores R., Di Serio F. (2012). Small RNAs containing the pathogenic determinant of a chloroplast-replicating viroid guide the degradation of a host mRNA as predicted by RNA silencing. Plant J..

[7] Adkar-Purushothama C.R., Brosseau C., Giguère T., Sano T., Moffett P., Perreault J.P. (2015). Small RNA derived from the virulence modulating region of the Potato spindle tuber viroid silences callose synthase genes of tomato plants. Plant Cell.

[8] Adkar-Purushothama C.R., Lyer P.S., Perreault J.P. (2017). Potato spindle tuber viroid infection triggers degradation of chloride channel protein CLC-b-like and Ribosomal protein S3a-like mRNAs in tomato plants. Sci. Rep..

[9] Bao S., Owens R.A., Sun Q., Song H., Liu Y., Eamens A.L., Feng H., Tian H., Wang M.-B., Zhang R. (2019). Silencing of transcription factor encoding gene StTCP23 by small RNAs derived from the virulence modulating region of potato spindle tuber viroid is associated with symptom development in potato. PLoS Pathog..

[10] Sänger H.L., Parthier B., Boulter D. (1982). Biology, structure, functions and possible origin of viroids. Nucleic Acids and Proteins in Plants II.

[11] Matoušek J., Kozlová P., Orctová L., Schmitz A., Pešina K., Bannach O., Diermann N., Steger G., Riesner D. (2007). Accumulation of viroid-specific small RNAs and increase in nucleolytic activities linked to viroid-caused pathogenesis. Biol. Chem..

[12] Gruner R., Fels A., Qu F., Zimmat R., Steger G., Riesner D. (1995). Interdependence of pathogenicity and replicability with potato spindle tuber viroid. Virology.

[13] Wright N.S. (1954). The witches’ broom virus disease of potatoes. Am. Potato J..

[14] O’Brien J., Raymer W.D. (1962). Transmission of potato spindle tuber virus in tomato. Am. Potato J..

[15] Diener T.O. (1971). Potato spindle tuber “virus”: IV. A replicating, low molecular weight RNA. Virology.

[16] Wang Y., Shibuya M., Taneda A., Kurauchi T., Senda M., Owens R.A., Sano T. (2011). Accumulation of Potato spindle tuner viroid-specific small RNAs is accompanied by specific changes in gene expression in two tomato cultivars. Virology.

[17] Owens R.A., Tech K.B., Shao J.Y., Sano T., Baker C.J. (2012). Global analysis of tomato gene expression during potato spindle tuber viroid infection reveals a complex array of changes affecting hormone signaling. Mol. Plant Microbe Int..

[18] Menda N., Strickler S.R., Mueller L.A. (2013). Advances in tomato research in the post-genome era. Plant Biotech..

[19] Peralta I.E., Spooner D.M., Knapp S. (2008). Taxonomy of wild tomatoes and their relatives (Solanum sect. Lycopersicoides, sect. Juglandifolia, sect. Lycopersicon; Solanaceae). Sys. Bot. Monogr..

[20] Blanca J., Canizare J., Cordero L., Pascual L., Jose D.M., Nuez F. (2012). Variation revealed by SNP genotyping and morphology provides insight into the origin of the tomato. PLoS ONE.

[21] Tomato Genome Consortium (2012). The tomato genome sequence provides insights into fleshy fruit evolution. Nature.

[22] Ranc N., Muños S., Santoni S., Causse M. (2008). A clarified position for solanum lycopersicum var. cerasiforme in the evolutionary history of tomatoes (solanaceae). BMC Plant Biol..

[23] Tanksley S.D. (2004). The genetic, developmental, and molecular bases of fruit size and shape variation in tomato. Plant Cell.

[24] Cong B., Barrero L.S., Tanksley S.D. (2008). Regulatory change in YABBY-like transcription factor led to evolution of extreme fruit size during tomato domestication. Nat. Genet..

[25] Pelham J. (1966). Resistance in tomato to tobacco mosaic virus. Euphytica.

[26] Bolger A., Scossa F., E Bolger M., Lanz C., Maumus F., Tohge T., Quesneville H., Alseekh S., Sørensen I., Lichtenstein G. (2014). The genome of the stress-tolerant wild tomato species Solanum pennellii. Nat. Genet..

[27] Singh R.P., O’Brien M.J. (1970). Additional indicator plants for potato spindle tuber virus. Am. Potato J..

[28] Singh R.P. (1973). Experimental host range of the potato spindle tuber ‘virus’. Am. Potato J..

[29] Nabeshima T., Matsushita Y., Hosokawa M. (2018). Chrysanthemum stunt viroid resistance in Chrysanthemum. Viruses.

[30] Matsushita Y., Aoki K., Sumitomo K. (2012). Selection and inheritance of resistance to Chrysanthemum stunt viroid. Crop Protect..

[31] Singh R.P. (1985). Clones of Solanum berthaultii resistant to potato spindle tuber viroid. Phytopathology.

[32] Sofy A.R., Mahfouze S.A., El-Enany M.A.M. (2013). Isozyme markers for response of wild potato species to Potato spindle tuber viroid egyptian isolate. World Appl. Sci. J..

[33] Palukaitis P. (2012). Resistance to viruses of potato and their vectors. Plant Pathol. J..

[34] Palukaitis P. (2014). What has been happening with viroids?. Virus Genes.

[35] Carbonell A., de Alba Á.E.M., Flores R., Gago S. (2008). Double-stranded RNA interferes in a sequence-specific manner with the infection of representative members of the two viroid families. Virology.

[36] Sano T., Nagayama A., Ogawa T., Ishida I., Okada Y. (1997). Transgenic potato expressing a double-stranded RNA-specific ribonuclease is resistant to potato spindle tuber viroid. Nat. Biotechnol..

[37] Yang X., Yie Y., Zhu F., Liu Y., Kang L., Wang X., Tien P. (1997). Ribozyme-mediated high resistance against potato spindle tuber viroid in transgenic potatoes. Proc. Natl. Acad. Sci. USA.

[38] Schwind N., Zwiebel M., Itaya A., Ding B., Wang M.B., Krczal G., Wassenegger M. (2009). RNAi-mediated resistance to Potato spindle tuber viroid in transgenic tomato expressing a viroid hairpin RNA construct. Mol. Plant Pathol..

[39] O’Brien J. (1972). Hosts of potato spindle tuber virus in suborder Solanineae. Am. Potato J..

[40] O’Brien J., Raymer W.D. (1964). Symptomless hosts of the potato spindle tuber virus. Phytopathology.

[41] Di Serio F., Martínez de Alba A.E., Navarro B., Gisel A., Flores R. (2010). RNA-dependent RNA polymerase 6 delays accumulation and precludes meristem invasion of a viroid that replicates in the nucleus. J. Virol..

[42] Aviña-Padilla K., Rivera-Bustamante R., Kovalskaya N.Y., Hammond R.W. (2018). Pospiviroid infection of tomato regulates the expression of genes involved in flower and fruit development. Viruses.

[43] Serrani J.C., Sanjuán R., Ruiz-Rivero O., Fos M., García-Martínez J.L. (2007). Gibberellin regulation of fruit set and growth in tomato. Plant Physiol..

[44] Ariizumi T., Shinozaki Y., Ezura H. (2013). Genes that influence yield in tomato. Breed. Sci..

[45] Dai M., Zhao Y., Ma Q., Hu Y., Hedden P., Zhang Q., Zhou D.-X. (2007). The rice YABBY1 gene is involved in the feedback regulation of gibberellin metabolism. Plant Physiol..

[46] Suzuki T., Ikeda S., Kasai A., Taneda A., Fujibayashi M., Sugawara K., Okuta M., Maeda H., Sano T. (2019). RNAi-mediated down-regulation of Dicer-like 2 and 4 changes the response of ‘Moneymaker’ tomato to potato spindle tuber viroid infection from tolerance to lethal systemic necrosis, accompanied by up-regulation of miR398, 398a-3p and production of excessive amount of reactive oxygen species. Viruses.

[47] Kwon J., Kasai A., Maoka T., Masuta C., Sano T., Nakahara K. (2020). RNA silencing-related genes contribute to tolerance of infection with potato virus X and Y in a susceptible tomato plant. Virol. J..

[48] Katsarou K., Mavrothalassiti E., Dermauw W., Leeuwen T.V., Kalantidis K. (2016). Combined activity of DCL2 and DCL3 is crucial in the defense against potato spindle tuber viroid. PLoS Pathog..

[49] Boller T., Felix G. (2009). A renaissance of elicitor: Perception of microbe-associated molecular patterns and danger signals by pattern-gecognition receptors. Annu. Rev. Plant Biol..

[50] Zheng Y., Wang Y., Ding B., Fei Z. (2017). Comprehensive transcriptome analyses reveal that potato spindle tuber viroid triggers genome-wide changes in alternative splicing, inducible trans-acting activity of phased secondary small interfering RNAs, and immune responses. J. Virol..

[51] Naoi T., Kitabayashi S., Kasai A., Sugawara K., Adkar-Purushothama C.R., Senda M., Hataya T., Sano T. (2020). Suppression of RNA-dependent RNA polymerase 6 in tomatoes allows potato spindle tuber viroid to invade basal part but not apical part including pluripotent stem cells of shoot apical meristem. PLoS ONE.

[52] Tsushima D., Adkar-Purushothama C.R., Taneda A., Sano T. (2015). Changes in relative expression levels of viroid-specific small RNAs and microRNAs in tomato plants infected with severe and mild symptom including isolates of Potato spindle tuber viroid. J. Gen. Plant Pathol..

[53] Więsyk A., Iwanicka-Nowicka R., Fogtman A., Zagórski-Ostoja W., Góra-Sochacka A. (2018). Time-course microarray analysis reveals differences between transcriptional changes in tomato leaves triggered by mild and severe variants of Potato spindle tuber viroid. Viruses.

[54] Nakahara K., Hataya T., Uyeda I. (1999). A simple, rapid method of nucleic acid extraction without tissue homogenization for detecting viroids by hybridization and RT-PCR. J. Virol. Methods.

[55] Hataya T. (2009). Duplex reverse transcription-polymerase chain reaction system to detect Potato spindle tuber viroid using an internal control mRNA and a non-infectious positive control RNA. J. Gen. Plant Pathol..

