# Supplementary material for: Tolerance Even to Lethal Strain of Potato Spindle Tuber Viroid Found in Wild Tomato Species Can Be Introduced by Crossing
Source: Plants (Basel). 2021 Mar 18;10(3):575. doi: 10.3390/plants10030575 (PMC8003082; doi:10.3390/plants10030575)
Supplement: Supplementary file 1 [file plants-10-00575-s001.pdf]

Supplementary materials

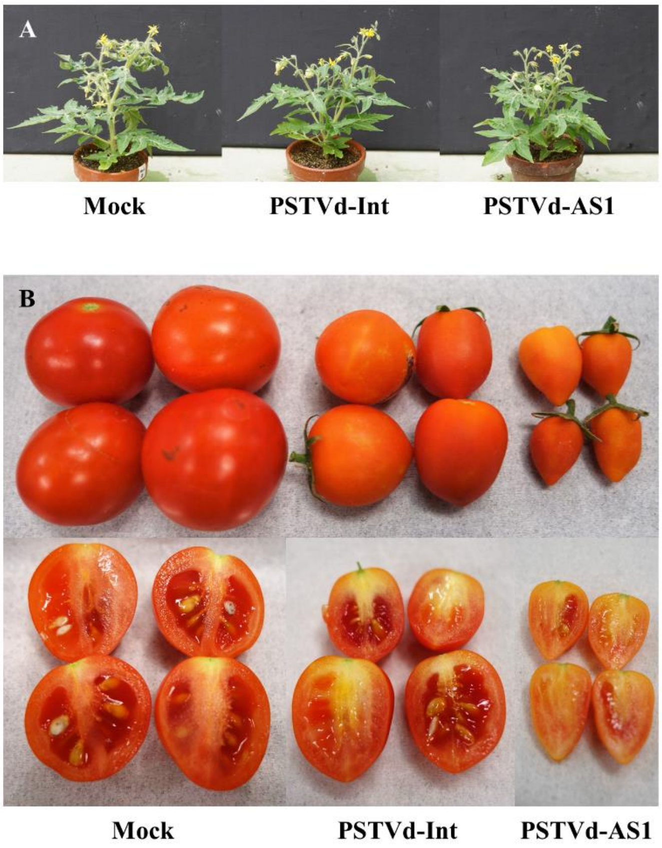

Figure S1 Symptoms in fruits of PSTVd-tolerant ‘Micro-Tom’ tomatoes. ‘Micro-Tom’ tomatoes showed clear disease symptoms only in fruits derived from PSTVd-infected plants. (A) ‘Micro-Tom’ tomatoes exhibited PSTVd tolerance to some extent, and did not clearly show morphological symptoms in stems and leaves at 6 weeks post-inoculation. (B) The fruits from PSTVd-infected plants became significantly smaller in size and lost their surface luster; the fruits from PSTVd-AS1-infected plants did not form any seeds. Differences in pathogenicity between PSTVd strains were remarkable with regard to fruit symptoms.

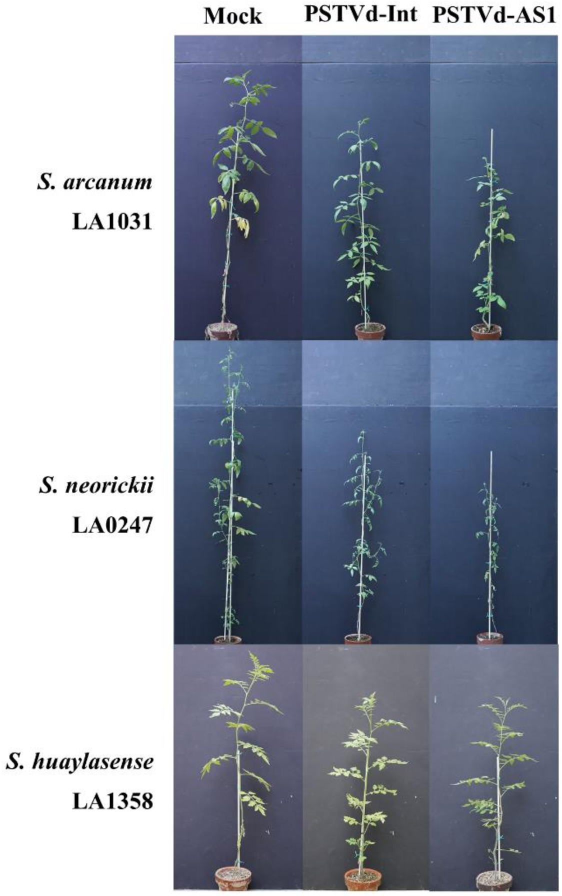

Figure S2 PSTVd-sensitive wild tomato relatives. In addition to the three species described in Figure 4, *S. arcunum* LA1031, *S. neorickii* LA0247, and *S. huaylasense* LA1358 were symptomatic during PSTVd infection. *S. arcanum* LA1031 plants exhibited stunting at 6 weeks post-inoculation (wpi). *S. neorickii* LA0247 showed stunt and leaf miniaturization at 6 wpi. *S. huaylasense* LA1358 exhibited stunting at 4 wpi, whereas plant growth tended to be suppressed under our test conditions. Differences in pathogenicity between PSTVd strains were remarkable in *S. arcanum* LA1031 and *S. neorickii* LA0247 plants

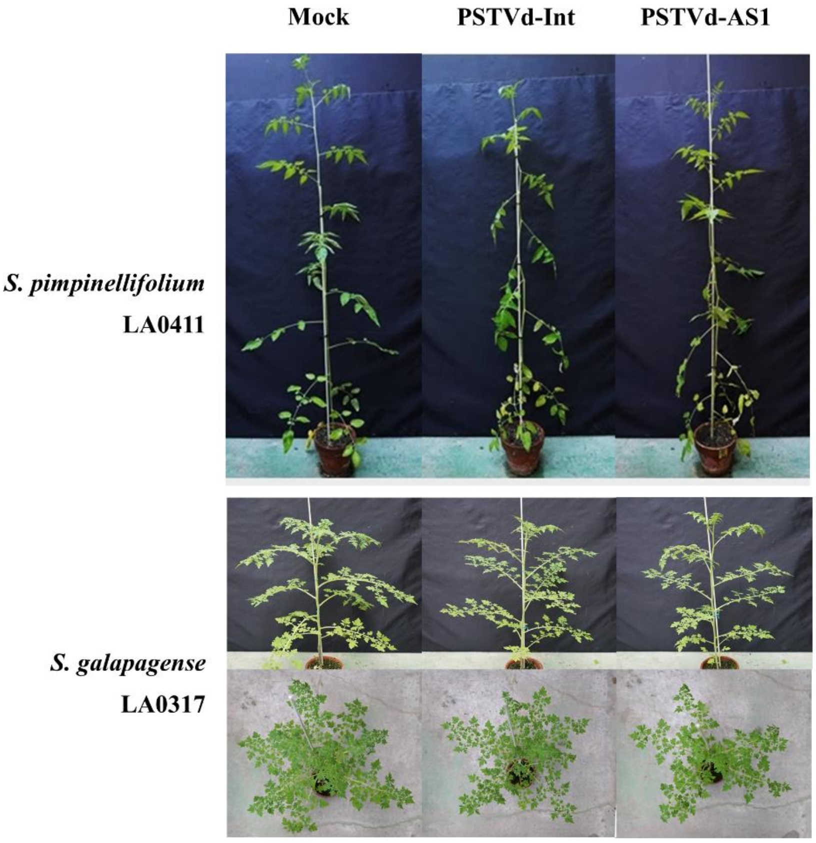

Figure S3 PSTVd-tolerant wild tomato relatives. In addition to the three species described in Figure 5, *S. pimpinellifolium* LA 0411 and *S. galapagense* LA0317 exhibited PSTVd tolerance regardless of PSTVd strain. *S. pimpinellifolium* LA0411 was asymptomatic during PSTVd infection at 6 weeks post-inoculation, similar to *S. pimpinellifolium* LA0373. *S. galapagense* LA0317 exhibited no obvious symptoms, except for poor growth, such as decreased number of branches and leaves.
